# Laparoscopic donor nephrectomy

**DOI:** 10.4103/0972-9941.19262

**Published:** 2005-10

**Authors:** Nitin Gupta, Pamposh Raina, Anant Kumar

**Affiliations:** Department of Urology and Renal Transplantation, Sanjay Gandhi Post Graduate Institute of Medical Sciences, Lucknow, Uttar Pradesh, India

**Keywords:** donor nephrectomy, transplant, warm ischemia

## Abstract

Of the various options for patients with end stage renal disease, kidney transplantation is the treatment of choice for a suitable patient. The kidney for transplantation is retrieved from either a cadaver or a live donor. Living donor nephrectomy has been developed as a method to address the shortfall in cadaveric kidneys available for transplantation. Laparoscopic living donor nephrectomy (LLDN), by reducing postoperative pain, shortening convalescence, and improving the cosmetic outcome of the donor nephrectomy, has shown the potential to increase the number of living kidney donations further by removing some of the disincentives inherent to donation itself. The technique of LLDN has undergone evolution at different transplant centers and many modifications have been done to improve donor safety and recipient outcome. Virtually all donors eligible for an open surgical procedure may also undergo the laparoscopic operation. Various earlier contraindications to LDN, such as right donor kidney, multiple vessels, anomalous vasculature and obesity have been overcome with increasing experience. Laparoscopic live donor nephrectomy can be done transperitoneally or retroperitoneally on either side. The approach is most commonly transperitoneal, which allows adequate working space and easy dissection. A review of literature and our experience with regards to standard approach and the modifications is presented including a cost saving model for the developing countries. An assessment has been made, of the impact of LDN on the outcome of donor and the recipient.

## INTRODUCTION

End stage renal disease (ESRD) patients have the option of life long maintenance haemodialysis, continuous ambulatory peritoneal dialysis (CAPD) or kidney transplantation. Kidney transplantation is the treatment of choice for a suitable patient with ESRD. The first cadaveric renal transplant was performed in 1945 and first live related renal transplant was performed in 1953.[1] Merril et al[2] performed the first successful living related (identical twin) renal transplant in Boston in 1954. The kidney for transplantation is retrieved from either a cadaver or a live donor. The shortage of organs remains the most important factor limiting kidney transplantation. Living donor nephrectomy has been developed as a method to address the shortfall in cadaveric kidneys available for transplantation.^3^ The surgical procedure for organ donation should be least disruptive, less painful and safe to the donor as live donors accept the inconvenience and risk of major surgery entirely for the benefit of the recipient.[4] Live donor transplants are associated with better short term and long term renal function compared to cadaveric transplantation.[5]

Live donor nephrectomy is a unique surgical challenge because it is performed on healthy donors. It is of great importance to keep the morbidity and mortality of live donors as low as possible and to harvest the kidney in optimal condition for transplantation. The traditional method of open live donor nephrectomy is associated with significant trauma to the thoracoabdominal wall with a long flank incision, more pain, possible pleural injury, pseudohernia, longer hospital stay, long term wound complications (hernia, hypoanaesthesia & chronic wound pain) and relatively long convalescence for otherwise healthy volunteer donors.[6,7] For a long time, live donor nephrectomy was performed only with an open surgical approach and thereby many possible kidney donors were reluctant to donate due to the morbidity associated with the technique of organ retrieval. To decrease the disparity between demand and organ supply and to reduce the donor morbidity, minimally invasive surgical techniques like mini-incision[8] and laparoscopic donor nephrectomy have been developed. Gill and colleagues[9] demonstrated feasibility of laparoscopic donor nephrectomy in a porcine model. First laparoscopic live donor nephrectomy (LLDN) was performed by Ratner etal[10] in 1995. Since then, the technique of LLDN has undergone evolution at different transplant centers and many modifications have been done to improve donor safety and recipient outcome.

Laparoscopic live donor nephrectomy minimizes the drawbacks of open live donor nephrectomy and makes the prospects of live donor nephrectomy more appealing to prospective donors by reducing post operative pain, shortening convalescence and improving the cosmetic outcome of donor nephrectomy. LLDN has shown the potential to increase the number of living kidney donations by removing some of the disincentives inherent to kidney donation itself.[17]

### Indications and contraindications of LDN

Virtually all donors eligible for an open surgical procedure may also undergo the laparoscopic operation. Various earlier contraindications to LDN, such as right donor kidney, multiple vessels, anomalous vasculature and obesity have been overcome with increasing experience.[3] The contraindications to kidney donation are similar in both open and laparoscopic procedures and focus on maintaining acceptable long term renal function in the donor.[11] Although a history of multiple abdominal surgeries in the donor may be considered a relative contraindication, a retroperitoneal laparoscopic approach in a donor with previous transperitoneal surgery and vice-versa may still be safely attempted.

### Standard operative techniques

Laparoscopic live donor nephrectomy can be done transperitoneally or retroperitoneally on either side. The operative technique of LLDN has undergone many refinements since it was first described. The approach is most commonly transperitoneal, which allows adequate working space and easy dissection.

### Left transperitoneal approach

**Figure 1:**
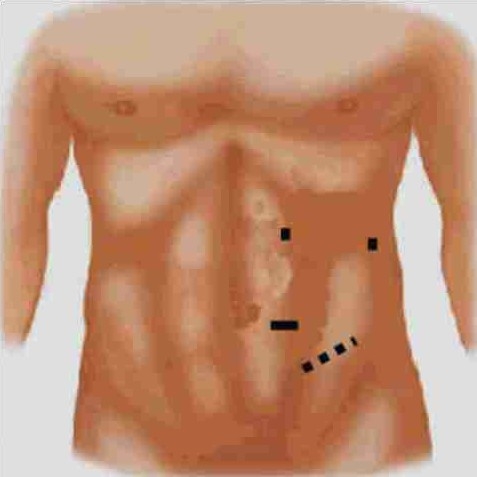
Standard ports for left transperitoneal donor

**Figure 2:**
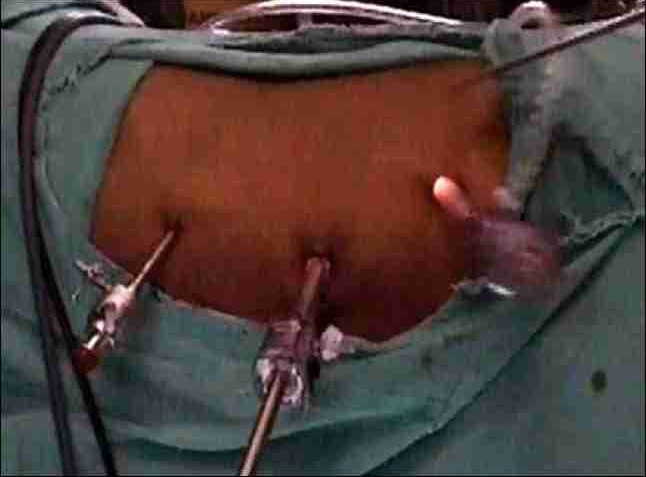
Standard ports for left transperitoneal donor

All the donors are hydrated overnight with 1.5 L of intravenous fluids. After general anaesthesia, a Foley's catheter is inserted. The donor is placed in a modified lateral decubitus position. Pneumoperitoneum is created by insufflation of carbon dioxide using a veress needle or after introduction of a laparoscopic 12–mm port at umbilicus by open Hassan technique. The umbilical port is primarily used as a camera port. Another 12–mm laparoscopic port is placed between umbilical port and anterior superior iliac spine (spinoumbilical port). A 5–mm port is placed in line with the camera port about 3 cm below the costal margin and 3 cm lateral to the midline. Fourth 5 mm laparoscopic port may be used for retraction, if needed and is placed 4 cm below the costal margin in anterior axillary line (Figure 1, 2).[12]

The lateral peritoneal reflection is incised along the line of Toldt from splenic flexure to pelvic inlet, and dissection is continued in the plane between Gerota's fascia and descending colonic mesentry. The left gonadal vein is followed up to the left renal vein. Before beginning the hilar dissection, 25 mg of mannitol is administered. At least 3–4 L of fluid is given during surgery to negate the effect of pneumoperitoneum.[12] The adrenal and lumbar veins are clipped and divided, and the renal vein is mobilized. The left renal artery is dissected at its aortic origin. If vasospasm is noted, the renal artery can be bathed in papaverine solution (30 mg/ml). The adrenal gland is separated from the upper pole of the kidney. After completing the hilar dissection, but before ligating the vessels, the patient is given another 25 mg of mannitol and some centers also give 20 mg of frusemide.[11] Vigorous intravenous hydration, mannitol/frusemide, and topical papaverine instillation on the renal artery help to minimize pneumoperitoneum pressure induced oliguria.[13,14] The gonadal vein is not divided near the renal vein, instead it is lifted up and using this as a guide, the dissection is continued on its medial border to prevent the compromise of the periureteric blood supply. No dissection is done between the gonadal vein and ureter. The ureteral packet with generous periureteric fatty tissue is dissected up to the level of iliac vessels and the gonadal vein is clipped and divided where it crosses over the ureter. The lower pole of kidney is elevated and the remaining posterior attachments divided with sharp and blunt dissection. The ureter is clipped distally and divided immediately above the level of iliac vessels, leaving its proximal end open for remainder of the dissection.

Some centers give 3000 units of low molecular weight heparin sulphate 5 min before ligating the vascular pedicle. A 15 mm laparoscopic organ retrieval bag (Endocatch, US Surgical, Norwalk, Connecticut, USA) is placed through a 6 cm pfannenstiel incision. By placing forceps between previously mobilized renal artery and vein, excellent exposure of renal hilum is possible. The renal artery and vein are sequentially ligated with a GI vascular stapler or 13mm Weck Clips (Hemolock, Weck Pilling, and USA). The kidney is then placed in the retrieval bag and carefully extracted from the pfannenstiel incision.

#### Our modification:

**Figure 3:**
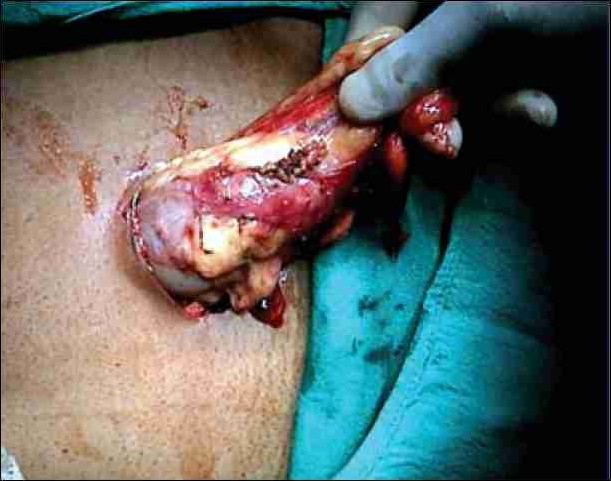
Two finger technique for graft retrieval

**Figure 4:**
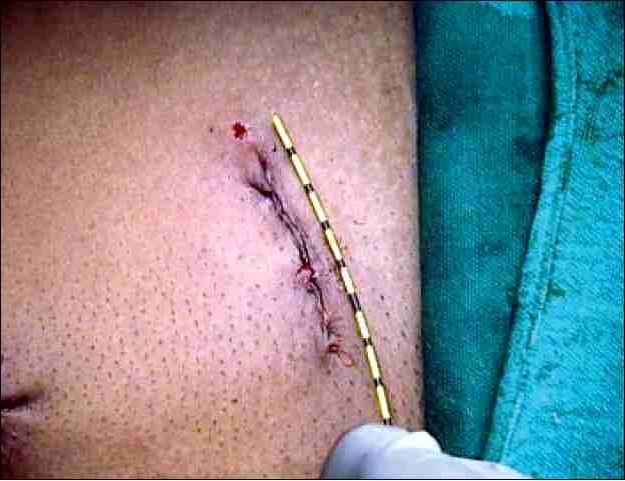
Five-centimeter final incision

We do not use the Endocatch organ retrieval bag or the stapler because of the high cost. A small amount of perirenal fat is left intact on the lateral and inferior border of the kidney and this is helpful in holding the graft during subsequent manual retrieval. We use 13mm Weck Clips for the ligating renal artery and vein & the kidney is extracted by a two finger technique.[19–12] A fan retractor is used to push the kidney down towards the iliac fossa, enabling it to reach the spinoumbilical port. A 5 cm incision is given extending the spinoumbilical port incision in an oblique course directing towards the symphysis pubis. The underlying muscle is split and peritoneum opened. Two fingers (index and middle) are inserted through the incision to hold the perirenal fat lateral to the kidney, and the graft gently retrieved through the 5 cm iliac fossa muscle splitting incision (Figure 3 and 4). The incision is closed with 2–0 polyglactin running suture. The pneumoperitoneum is recreated to check for hemostasis at a lower intraabdominal pressure setting. A 14 F closed drain is kept in the renal fossa, which is removed next morning. The carbon dioxide is evacuated from the abdomen and all the trocar sites and skin incision is closed. Advantage of this incision is that, it only extends 3.8cm from the existing port incision. The muscle is only split, not cut and so it is less painful and always hidden by the usual dress worn in this part of the country.

#### Right transperitoneal approach:

The transperitoneal laparoscopic approach may be used similar to that on the left side. Modifications have been made in right LLDN to compensate for shorter vessels and to prevent thrombotic complications. In the recipient, the iliac vein is completely mobilized by dividing all of its posterior branches to facilitate making a tension free anastomosis.[11] The dissection of interaortocaval space to allow the division of renal artery at its origin from aorta has been recommended by some investigators.[7] This maneuver allows for the division of right renal artery at its origin, rather than at lateral border of vena cava. Another technique to gain arterial length involves mobilizing the vena cava by dividing the lumbar veins.[15] This allows the vena cava to be rolled anteriorly and the kidney to be placed in a “flipped” position, increasing exposure of the right renal artery at its origin from the aorta. The kidney is then retracted laterally and endovascular stapling device placed parallel to aorta at the origin of right renal artery, thus allowing for the maximization of arterial length.

Insertion of an Endo-GIA stapler through the port positioned in the right lower abdominal quadrant allows control of the renal vein at its junction with vena cava, preserving the maximum possible length of renal vein.[16] To maximize the length of right renal vein, one of three modifications is done.[11] The first involves use of a TA stapler which fires two staple lines without cutting. The vein is subsequently cut flush with the staple line to gain extra length. The stapler is introduced through the lateral trocar site, in a plane parallel to the inferior vena cava. The parallel orientation of the stapler in relation to the vena cava results in preservation of additional right renal vein length.[3] The second modification involves open surgical division of right renal vein. A 5–6 cm right subcostal incision is made. After laparoscopic dissection of right kidney, a Satinsky clamp is placed on the inferior vena cava just medial to the origin of the right renal vein. Alternatively, a laparoscopic Satinsky clamp can be used to obtain a cuff of vena cava, which is then repaired intracorporeally.[17] A third modification is used in case of a short renal vein and a graft of the recipient's saphenous vein can be used to reconstruct the renal vein.

#### Left retroperitoneal approach:

The donor is given general anaesthesia, catheterization done with a Foley catheter and is placed in an overextended flank position.[18] The first incision is given just below the tip of 12^th^ rib. The underlying muscles are split till the thoracolumbar fascia is seen, which is incised sharply and retroperitoneal space is entered. Blunt finger dissection of pararenal fatty tissue is done to separate the fatty tissue from the abdominal wall to create an initial small retroperitoneal space for the placement of dilating balloon. Retroperitoneal space is now created by a PBD-1000 balloon device (Tyco, USA). A 12 mm trocar replaces the balloon device and it is used as a camera port. Two more trocars (one 12mm & other 10 mm) are placed under vision. During operation, a pressure of about 10–12 mm of Hg is enough to create a sufficient retroperitoneal working space.[25] Hook monopolar scissors or harmonic scalpel are used for dissection.

After identifying the psoas muscle, one of the most important landmarks, Gerota's fascia is incised laterally, and retroperitoneal space is freed in cranial and caudal direction. It is important to keep the psoas muscle horizontally on the video screen to remain oriented. The ureter is identified in the area of medial line of psoas muscle, is carefully freed, care being taken not to dissect the periureteric sheath and hence damage the nutritive vessels of the ureter, which will result in necrosis after transplantation. Perirenal fatty tissue is identified and renal vessels are freed of surrounding lymphatic tissue. Gentle and meticulous dissection is necessary to avoid injuries to the renal vessels. The gonadal vein is dissected adjoining the renal vein & may be clipped and divided if required. The renal artery is dissected and freed at its origin from the aorta. The adrenal vein is identified, dissected near the renal vein, clipped and divided. The dissection proceeds to separate the adrenal gland from the upper pole of left kidney. The surrounding fatty tissue is freed from the renal capsule. The gonadal vein is clipped and divided at the pelvic brim if not already divided. The ureter is clipped distally and transected. The kidney can be retrieved by any of the incisions: muscle splitting lumbotomy incision, illiac fossa incision inferior to the anterior port or by a pfannenstiel incision after creating space extraperitoneally.

#### Our modification:

**Figure 5:**
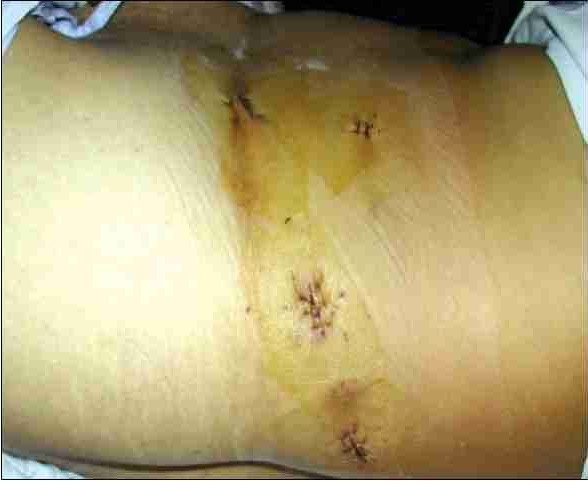
Site for ports for left retroperitoneal donor

We use a balloon made by two middle fingers of a glove, pulled one over the other and fixed on a plastic tube as described by Gaur et al.[19] This device is cost effective and takes only few minutes to create. 450– 500 ml of saline is infused into the balloon and kept for 10 min. Further, the retroperitoneal space is created by blunt dissection under vision. The first port is used as a camera port with 2 other ports being inserted under vision. 2^nd^ port is placed 2–3 cm behind the first post below the 12^th^ rib and a third port 10 mm anterior to the first port (Figure 5). After dissection of the main renal vessels, the gonadal vein is identified and traced down till the lower ureteric end. We do not clip the gonadal vein near the renal vein in order to preserve the fatty tissue in the golden triangle (area between the ureter, gonadal vessel and lower pole of kidney). The renal vessels are dissected till the aorta on the left side and the IVC on the right side. This is absolutely necessary to gain the full length of the vessels. The dissection is continued with preparation of the lateral circumference of the kidney. The surrounding fatty tissue is removed from the kidney except at the lower pole where we tend to preserve the fat. The ureter are renal vessels are clipped with hem-o-lock clips and cut.

#### Right retroperitoneal approach:

Gill et al[20] demonstrated that the technical difficulties during right LDN may be overcome using a retroperitoneal approach (Table 4).The advantages of right retroperitoneal approach are: direct organ exposure, reduced interference from intra-abdominal viscera, easier management of right renal vasculature, better and control of right renal artery in a retrocaval location and adequate renal artery length. A reusable 10 mm fan retractor is used for kidney retraction with decreased risk of sub-capsular haematoma or parenchymal laceration. The antero-medial peritoneal attachments of the kidney are kept intact until the end of operation to prevent the posterior displacement of kidney. Preservation of peri-ureteral fatty tissue prevents ureteral complications. A modified muscle splitting Gibson incision is used to retrieve the kidney. Adequate exposure in retroperitoneal laparoscopic surgery is a must to complete this challenging operation.

We prefer to harvest the right kidney in those cases where it has some pathology (comparatively low GFR, stone, cyst, scar etc) and leave the better functioning kidney with the donor. Right LDN is done only retroperitoneoscopally at our centre as this gives better access to the hilum with good length of vein and artery.

Our modification of right retroperitoneal approach Initial part of the procedure is similar to that described above and just before the removal of the graft, an incision is made between the 1^st^ and anterior ports. Only superficial layer of the muscles is incised and a sponge is kept to prevent air leak. The posterior port is used to clip and cut the renal artery. Subsequently, an incision is given, the anterior port is removed and an open surgical Satinsky clamp is inserted and applied on the IVC under vision. The vein is divided with a cuff of the IVC, the graft is retrieved and the IVC is sutured using a long needle holder. The final knot is placed by throwing the knot and using the right angle to guide and tighten it as there is no space for the hand to go in.

The limited working space makes dissection difficult due to repeated fogging of lens and retroperitoneal fat keeps coming in the way. We could overcome this difficulty by inserting a fourth port and using a 5 mm fan retractor to retract the fat and at the same time giving gentle traction at the kidney to facilitate hilar dissection. However, retraction of the kidney with 10mm/5mm retractors results in small subcapsular hematomas with could be seen in 60% of our patients. They are common on the posterior aspect near the hilar area and near the upper pole where maximum retraction is used while dissecting the hilar structures and mobilizing the kidney. These hematomas did not affect the functional graft outcome.

#### Hand assisted laparoscopic donor nephrectomy:

Hand assisted LDN has been described both transperitoneally and retroperitoneally. Ruiz-Deya et al^22^ are of the view that potential advantages of hand- assisted LDN over pure LDN include shorter operating and warm ischemia time, better exposure of renal hilum, an enhanced tactile sensation, safer vessel transection, and rapid graft retrieval. The disadvantages of this technique include longer duration of ileus and hence delayed recovery of bowel function, a longer convalescence time and impaired cosmetic result because of epigastric abdominal incision.

Sundqvist et al[23] compared hand assisted retroperitoneoscopic LDN with open and pure LDN and found that hand assisted retroperitoneoscopic live donor nephrectomy is safe, quick and the donors experience little pain. Bleeding is the most common reason for conversion to open surgery. When hand assisted technique is used, the surgeon's fingers can compress the bleeding vessel immediately. Hand assistance preserves tactile sense during surgery. This increases safety, in addition to shortening the duration of operation and the learning curve. With the introduction and development of new surgical methods, it is also important that the technique is safe and easy to perform especially for surgeons at a smaller transplant centre or those with limited experience. A recent study by El-Galley etal,[24] comparing hand assisted and pure laparoscopic technique, demonstrated similar results in terms of complications, graft function, early recovery, and time to return to normal physical activity.

#### Results in terms of donor outcome:

At many centers, the number of individuals willing to undergo LLDN today would not have donated a kidney if open surgery was the only option available for organ donation. This is so because LLDN offers decreased postoperative pain, marked cosmetic benefit, and better convalescence compared to open donor nephrectomy.

#### Operative time:

In most reported experiences, the operating time at the beginning of the LDN learning curve was generally longer than for open live donor nephrectomy.[25] In a meta-analysis of published reports regarding the current status of LDN, the operating time was significantly longer (183 to 340 vs 95–260 min, *P* < 0.05) in LDN series compared with open donor nephrectomy series.[26] However, with increasing experience, it decreases and tends to plateau after about 25 cases.[27] Our operative time for transperitoneal LDN is 150 min and sometimes we have finished the operation in 120 min. Our operative time for retroperitoneoscopic LDN is about 190 min. (Tables 1 and 4)

**Table 1:** The Donor

Investigator		No. of Patients	WIT(min)	EBL (ml)	OR time (min)	Open conversion (%)	Hosp.Stay (days)
Ratner^7^	LDN	70	—	266	230	—	3
	ODN	20	—	393	183	—	5.7
Rawlins^27^	LDN	100	2.3	102	231	1	3.3
	ODN	50	—	193	209	—	4.7
Simforoosh^36^	LDN	40	6.6	—	251	2.5	2.2
	ODN	40	2.09	—	135	—	2.1
Jacobs^33^	LDN	738	2.81	128	202	1.6	2.6
EL Galley^24^	LDN	28	3	200	306	0	2
	ODN	55	2	320	163	—	3
SGPGIMS	LDN	300	4.5	85	180	12	3.14
	ODN	1000	2	220	110	—	5.7

WIT = Warm Ischemia Time

EBL= Estimated Blood Loss

OR time = Operation Room Time

**Table 2:** Donor outcome–quality of life parameters

	Ratner^17^		Odland^30^		lowers^32^	
Parameters	LDN	ODN	LDN	ODN	LDN	ODN
n	25	37	26	30	70	65
PO intake (d)	—	—	—	—	1.7	3.2
LOS (d)	2.9	5.5	1.0	2.5	1.2	2.5
Analgesic usage (mg)	4.2	11.8	1.0	2.5	1.2	2.5
Driving (wks)	1.9	3.2	—	—	1.6	4.5
Caring for home (wks)	1.8	4.5	—	—	1.3	3.8
Full activity (wks)	—	—	1.4	3.6	—	—
Return to work (wks)	4.4	6.3	2.7	5.3	2.3	7.4
Exercising (wks)	3.6	9.4	—	—	—	—

LOS = Length of Hospital Stay

**Table 3:** Recipient outcome

Variables	SGPGIMSSoulsby^34^			Simforoosh^36^	
	ODN n=1000	LDN n=300	LDN n=54	ODN n=40	LDN n=40
S. Creat. (preop.)	5.1	5.34	—	—	—
S. Creat. (day 1)	2.36	2.56	—	—	—
S. Creat. (day 3)	1.71	1.63	1.68	1.46	1.91
S. Creat. (day 7)	1.69	1.72	1.67	—	2.0
S. Creat. (1M)	1.25	1.28	—	—	1.7
S. Creat. (3M)	1.35	1.42	1.19	1.37	1.32
S. Creat. (6 M)	1.46	1.41	1.22	1.43	1.23
S. Creat. (1y)	1.51	1.52	1.26	—	—
Return to normal S.					
Creat. (day2) %	56%	51%	—	—	—
Return to normal S.					
Creat. (day 5) %	78%	76%	—	—	—
Rejection (3 M) %	27.5%	25.4%	22	—	—
ATN (3 M) %	12.9%	13.5%	—	—	—

**Table 4:** Comparison between Transperitoneal and retroperitoneal LDN

	SGPGIMS Retroperitoneal	Gill et al^20^ Transperitoneal	Buell et al^41^ Retroperitoneal	Transperitoneal
Patients (n)	33	35	28	96
Side L:R	25:8	35	0:28	0:96
Operative time (min)	192 (150–330)	160 (120–210)	190.4	235
Blood Loss (ml)	100 (50–500)	80 (50–150)	107.8 (50–400)	139.2 (20–1200)

#### Blood loss:

LDN and open donor nephrectomy are comparable in the estimated blood loss and postoperative transfusion requirement.[14,28–30] In fact, most series reported lesser blood loss in the laparoscopic group compared to the open group. A comparison of transperitoneal left LDN and retroperitoneal right LDN revealed significantly less blood loss in the retroperitoneal LDN group.[20] We have found no significant difference in blood loss in the transperitoneal and retroperitoneal LDN; however blood loss in the LDN has been significantly reduced compared to ODN as seen in many other series (Tables 1 and 4).[24,27]

### Open conversion

The reported frequency of open conversion during LDN ranges from 0% to 13%.[30] The most common causes of conversion to open donor nephrectomy are intraoperative hemorrhage or vascular injury (65%), difficult kidney exposure or obese donor (20%), vascular stapler malfunction (12%), and pneumoperitoneum loss (3%).[26] Sometimes it is difficult to proceed due to dense adhesions around the renal hilum and prominent lymphatics (Table 1).

### Postoperative pain:

Reduced postoperative pain and recuperative time, the major advantages of LDN, have been demonstrated in several studies. Ratner et al[31] concluded that the amount of parenteral analgesia given to donors after LDN was significantly lower than after open donor nephrectomy (*P* < 0.05). Similar results have been found in other series (Table 2).[30,32]

### Hospital stay and convalescence:

Comparing LDN to open living donor nephrectomy, the hospital stay, resumption of oral intake, the time to return to home have been found to be significantly favorable to the LDN group (Table 1).[30–32]

## Quality of life parameters

Several series have addressed donor quality of life following LDN and ODN. It has been shown that LDN resulted in a shorter time until patients were able to drive, take care of the home, and return to full activity, work and regular exercise.[31,27,30,32] In comparison of pure laparoscopic live donor nephrectomy *vs* hand-assisted laparoscopic live donor nephrectomy, the former showed a shorter time to return to normal physical activity and work (Table 2).[24]

## Recipient's outcome

### Warm ischemia time (WIT):

This represents a major concern as it has always been slightly longer in the laparoscopic group when compared with open donor nephrectomy due to the longer extraction time. It has been thought that any increase in this would translate into a poor graft function. This notion has been disproved by various studies suggesting no bearing of this small difference on the recipient outcome. The warm ischemia time during LDN may range between 95 and 300 seconds.[27,28] At the University of Maryland, in 738 cases performed during a 6-year period, the warm ischemia time was 169 ± 90.8 seconds.[33] The warm ischemia time can be reduced with increasing experience, as shown by Rawlins et al. (3.3 min in first 25 LDN cases *vs* 1.8 min in the most recent 25 LDNs (*P* < 0.001).[27] Similar result has been shown by Soulsby et al.[34] The effects of WIT on delayed graft function was assessed extensively by Jacob et al, where rate of decline in serum creatinine (SCr) concentration, SCr in the first 10 days, changes in SCr at 3 months, acute rejection rate, biopsy-proven chronic allograft rejection and graft survival were assessed according to duration of WIT.[33] Analysis was made by comparing WIT d” 3 *vs* e” 3 minutes and WIT d” 5, 5–10, and e” 10 min. Prolonged WIT did not appear to have an effect on early graft function or the rate of decline in serum creatinine during the first 3 months post-transplantation.

We evaluated the impact of warm ischemia time in LDN. It did not correlate with the incidence of delayed graft function, acute rejection or allograft or recipient survival (Tables 1 and 3). In two patients it took 11 min and 14 min to retrieve kidneys, but both the kidneys worked well and had diuresis in the immediate post-operative period

#### Vessel and ureteral length:

There have been concerns for vessel and ureteral length which could be harvested in LDN as it would have a direct bearing on the recipient operation. In a randomized controlled trial[35] studying the structural and functional aspects of LDN and ODN it was shown that the left renal vein (p = 0.14) and left renal artery length (P = 0.38), right renal vein (p = 0.38) and artery length (p = 0.33) were similar. Ureteric length was significantly greater in the LDN group for both left and right nephrectomy. This study demonstrated that LDN yielded kidneys that were structurally and functionally equivalent to those acquired by the open operation.

#### Graft function:

Several clinical trials, have shown, no significant difference in the serum creatinine level between open and LDN at 3 days, 30 days, and 3 months after transplantation.[36] The longer warm ischemia time in the laparoscopic group has been shown not to affect the long-term graft outcome. In a systematic review of 24 comparative studies for both ODN and LDN, the trend was for values to start at approximately 4.0 to 5.0 mg/dl on day 1 after surgery but to drop to approximately 1.5 by day 7 and to stabilize at approximately that level thereafter.[37] At a follow-up of 6 months, Rawlins et al. confirmed no significant difference in serum creatinine level in LDN *vs* open donor nephrectomy group (1.64 *vs* 1.48 mg/dL, respectively, p =0.26).[27]

In a comparison between open donor nephrectomy and LDN in patients with multiple renal arteries, the postoperative serum creatinine level after 1 year of follow-up has been found to be similar (1.3 mg/dL *vs* 1.3 mg/dL, respectively, p = 0.9), and the graft survival rate (87.5% *vs* 87.5%, respectively) has also been similar.[37] Regardless of the procurement technique (open *vs* laparoscopic), live donor kidney transplantation was associated with a delayed graft function rate of 5% to 10%.[38]

An analysis of risk factors potentially affecting delayed graft function after laparoscopic live donor nephrectomy had shown that recipient age, donor/recipient sex relationship, unrelated highly mismatched donors, and cold/total preservation time to be associated with impaired renal function recovery. However, the laparoscopic approach was not related to delayed graft function (Table 3).[^44^]

#### Early graft rejection:

A systematic review of reported LDN series found no significant differences between the rate of acute rejection after LDN (2% to 30%) and open donor nephrectomy (0% to 32%).[26] Similar results have been reported from University of Maryland with more than 700 cases wherein early graft rejection occurred in 3% of cases (Table 3).[33]

#### Ureteral complications:

In initial LDN series, ureteral injuries occurred more frequently than during open donor nephrectomy (0% to 11% *vs* 0% to 6%, respectively).[26] Subsequent technical modifications (e.g. preservation of the peri ureteral tissue allowing adequate ureteral blood supply) have reduced the incidence of such complications. Refinements in surgical technique allowed Ratner et al. to reduce an initial 9.1% incidence of ureteral complications in the first 110 cases to 3% in the last 100 cases.[7] University of Maryland has reported ureteral complications initially about 7% which have gradually decreased to about 2.5%.[33]

#### Cost analysis:

Wolf et al[28] reported a higher operating cost in LDN series (73% greater) compared with open donor nephrectomy. However, when we consider the total cost of the procedure including hospital stay, analgesic requirement, loss of days of work and the need for supportive care, LDN fares better compared to the ODN, not adding the additional benefit of better cosmesis.[39] Presently, at our institute, ODN costs about Rs. 15750 while LDN costs Rs. 22500. This constitutes the total cost incurred during stay in the hospital.

## CONCLUSIONS

Since the start of live donor program in 1953, there has been a constant effort by the health care professionals to reduce the donor morbidity and improve their outcome. LDN has come as an answer to this, especially with refinements in equipment and surgical techniques. It provides all the benefits of minimal invasive surgery to the most unique patient that is the donor while maintaining results equivalent to the ODN in terms of graft function and recipient outcome. LDN has now stood the test of time. The results are equal and morbidity is less, hence its increasing popularity and becoming the standard of care.
